# Anthropometric and Laboratory Variables Related to Weight Loss—Comparison of Heart Failure Patients with Tumor Patients and Control Population

**DOI:** 10.3389/fnut.2017.00018

**Published:** 2017-05-11

**Authors:** Tomislav Letilovic, Radovan Vrhovac, Željko Krznarić

**Affiliations:** ^1^University Hospital Merkur, Department of Internal Medicine, Division of Cardiology, University of Zagreb School of Medicine, Zagreb, Croatia; ^2^University Hospital Centre Zagreb, Department of Internal Medicine, Division of Hematology, University of Zagreb School of Medicine, Zagreb, Croatia; ^3^University Hospital Centre Zagreb, Department of Internal Medicine, Division of Gastroenterology, Hepatology and Nutrition, University of Zagreb School of Medicine, Zagreb, Croatia

**Keywords:** weight loss, heart failure, tumor, C-reactive protein, albumin, hemoglobin, anthropometry

## Abstract

**Background:**

Body weight loss is an important feature of heart failure (HF) and tumors. It is related to both reduced survival and adverse reactions to therapy in both of these conditions. The mechanisms of body weight loss in patients with HF and tumors are considered to be similar. Yet, studies comparing those two populations are generally lacking. The aim of this study was to compare anthropometric and laboratory data, related to weight loss, between patients with chronic HF and patients with different tumors as well as control population.

**Methods:**

Laboratory and anthropometric data on 143 consecutive patients with chronic HF and malignant diseases as well as data for 20 controls were collected.

**Results:**

Patients with HF had lower levels of C-reactive protein (CRP) and albumin compared to controls. Anthropometric measurements revealed lower body mass index (BMI), muscle strength, mid-arm circumference, and waist circumference in patients with HF compared to controls. Measurements of biceps, triceps, subscapular, and suprailiac skinfolds were also lower in HF group. Compared to solid tumor group, HF patients had lower levels of CRP and higher levels of hemoglobin. Solid tumor patients had lower values of BMI and subscapular skinfold thickness, as well as higher muscle strength compared to HF group. Finally, compared to patients with solid hematological tumors, HF group had lower levels of albumin, lower muscle strength, as well as lower mid-arm circumference.

**Conclusion:**

We found differences in anthropometric and laboratory features, related to weight loss, in patients with HF compared to control population that were expected. On the other hand, observed differences in HF group compared to patients with various tumors could imply different pathophysiological mechanisms of weight loss between those groups. Such data could serve as a cornerstone for studies with larger numbers of patients and deeper pathophysiological insight.

## Introduction

Weight loss is a common finding in patients with chronic heart failure (HF) and tumors, as well as in other chronic diseases. Significant weight loss, usually defined as cachexia, affects approximately five million people in the United States of America annually (1) and results in two million deaths (2). It is also related to adverse reactions to various therapeutic modalities (3, 4).

Pathophysiological mechanisms responsible for weight loss in chronic HF and tumors are regarded similar (5, 6). The same mechanisms responsible for weight loss are responsible for some important laboratory and anthropometric features that can be found in patients suffering from chronic HF or tumor diseases. Several studies have found important differences in anthropometric measurements between patients with chronic HF (7) or tumors (8) and control population. Differences in several laboratory parameters were also found. Among them C-reactive protein (CRP), hemoglobin, and albumin are best studied. Their values were, therefore, incorporated in the recent definitions of the most pronounced form of weight loss called cachexia (6, 9). To date, there are no studies that compared anthropometric and laboratory features of patients with chronic HF and tumor diseases.

Principal aim of our study was to investigate possible differences in anthropometric and laboratory features, related to weight loss, in patients with chronic HF compared to patients with various tumors and control population. We gathered clinical data from 143 consecutive patients with malignant diseases or chronic HF hospitalized in a single institution. Patients with malignant diseases were further subdivided into group suffering from solid tumors and a group with hematological malignancies. We also gathered same data for 20 controls. We then compared anthropometric and laboratory findings of patients with chronic HF with patients in both tumor groups and with control group.

## Patients and Methods

### Patients

The study was conducted at the Department of Internal Medicine of University Hospital Merkur in Zagreb. Data were collected from May 2011 until May 2012 in consecutive patients who were eligible for entry. A diagnosis of either solid hematological tumors (non-Hodgkin lymphoma, Hodgkin disease), other solid tumors of any site, or chronic HF of any etiology was mandatory. Diagnosis of malignant disease had to be proven with adequate histopathological sample. Diagnosis of HF was made according to the guidelines criteria (10). Control population consisted of persons without proven malignancy or chronic HF.

Exclusion criteria were: age less than 18 years, starvation defined as deliberate or unintentional reduced food consumption despite preserved appetite, malabsorption defined as documented or suspected disease related to malabsorption, diarrhea defined as having three or more loose or liquid stools per day or as having more stools than is normal for a given patient, active thyroid disease, depression or other severe psychiatric disease, chronic obstructive pulmonary disease, chronic kidney disease stage ≥3, myocardial infarction in less than last 12 weeks, liver insufficiency defined by clinical or laboratory signs of its synthetic or metabolic dysfunction or a documented cirrhosis, neuromuscular diseases, and alcohol or drug abuse.

The study received previous local ethical board approval and was conducted according to the Declaration of Helsinki and its subsequent amendments. All patients gave written informed consent before participation in the study.

### Evaluation of Patients

Complete history and full physical examinations were performed at baseline. Baseline blood analyses included complete blood count and complete biochemistry (including values of hemoglobin, CRP, and albumin). All measurements were done according to the protocol of institutional laboratory.

Anthropometric measurements were done as follows. Muscle strength was obtained using handgrip dynamometer. Three consecutive measurements at least 5 s apart were done. Highest of the measurements was used for analysis. Measurements of skinfolds (biceps, triceps, subscapular, and suprailiac) and circumferences (mid-arm, waist, and hip) were done using previously published protocols (7). All measurements were done three times at each site. Calculated mean value was used for analysis. Body mass index (BMI) was calculated as weight in kilograms divided with squared height in meters.

### Statistics

The results were expressed as the mean ± SD or as a proportion of the total number. Mann–Whitney test was used to test the equality of continuous variables. A *p*-value < 0.05 was considered statistically significant. All statistics were performed with the StatView™ statistical program, version 5.0.1 (SAS Institute, Cary, NC, USA).

## Results

### Baseline Clinical Characteristics of Patients

One hundred forty three (86 males) consecutive patients and 20 (11 males) controls were enrolled. Chronic HF was diagnosed in 45 patients (33 had ischemic cardiomyopathy, 7 had dilated cardiomyopathy, and 5 had valvular cardiomyopathy). Out of them 4 (9%) were in NYHA I stage, 16 (36%) were in NYHA II stage, 18 (45%) were in NYHA III stage, and 7 (20%) were in NYHA IV stage. Fifty-five patients were diagnosed with solid hematological tumor (45 had diagnosis of non-Hodgkin lymphoma and 10 had diagnosis of Hodgkin disease). Forty-three had solid tumor of various sites (19 hepatocellular cancer, 8 colorectal cancer, 8 pancreatic cancer, 3 billiary duct cancer, 2 gastric cancer, and 3 cancer of unknown primary site). Initial evaluation revealed that patients with chronic HF were significantly older (mean age 73.9 ± 10.5) than patients with solid tumors (mean age 62.9 ± 12.6; *p* = 0.0001), hematological diseases (mean age 56.9 ± 14.6; *p* = 0.0001), and control group (mean age 65.1 ± 12.7; *p* = 0.007). We found no significant difference in the amount of weight loss, intensity of weight loss (kilograms/month), or prevalence of significant weight loss (defined as >5% loss in 12 months or less) between the disease groups.

### Anthropometric and Laboratory Findings—Chronic HF Patients vs. Control Population

There was no significant difference in hip circumference in chronic HF patients compared to control population. All other anthropometric measurements were found to be significantly lower in chronic HF patients compared to controls.

Laboratory measurements revealed significantly higher levels of CRP (10.0 ± 11.6 vs. 4.3 ± 5.5 mg/L; *p* = 0.01) and lower levels of albumin (3.8 ± 0.5 vs. 4.4 ± 0.4 g/dL) in chronic HF patients compared to controls. Differences in anthropometric variables and laboratory parameters, related to weight loss, between HF group and controls are shown in Table 1.

**Table 1:** **Differences in anthropometric and laboratory variables, related to weight loss, between HF group and controls**.

	HF	Controls	*p*-Value
Body weight (kg)	75.9 ± 15.4	84.4 ± 16.7	0.04*
Body mass index– (kg/m^2^)	26.5 ± 4.8	28.4 ± 8.0	0.04*
Grip strength (kg)	17.2 ± 9.7	26.8 ± 8.6	0.0005*
Mid-arm circumference (cm)	27.9 ± 3.7	30.4 ± 3.4	0.009*
Waist circumference (cm)	94.0 ± 11.2	100.0 ± 14.7	0.03*
Hip circumference (cm)	100.1 ± 8.8	100.4 ± 10.1	NS
Biceps skinfold (cm)	0.75 ± 0.44	1.0 ± 0.4	0.008*
Triceps skinfold (cm)	1.23 ± 0.50	1.48 ± 0.45	0.04*
Subscapular skinfold (cm)	1.43 ± 0.48	1.87 ± 0.5	0.002*
Suprailiac skinfold (cm)	1.28 ± 0.52	1.56 ± 0.51	0.04*
C-reactive protein (mg/L)	10.0 ± 11.6	4.3 ± 5.5	0.01*
Hemoglobin (g/dL)	13.3 ± 2.2	13.7 ± 1.4	NS
Albumin (g/dL)	3.8 ± 0.5	4.3 ± 0.4	0.0001*

*Values are given as a mean ± SD*.

*p < 0.05—Mann–Whitney U test.

*HF, heart failure; NS, not significant*.

### Anthropometric and Laboratory Findings—Chronic HF Patients vs. Solid Tumors Patients

Chronic HF patients were found to have higher BMI (26.5 ± 4.8 vs. 24.4 ± 4.7 kg/m^2^; *p* = 0.04) and higher measurements of subscapular skinfold (1.43 ± 0.48, vs. 1.24 ± 0.54 cm; *p* = 0.04) when compared to patients in solid tumor group. On the other hand, HF patients were found to have lower muscle strength (17.2 ± 9.7 vs. 24.7 ± 9.6 kg; *p* = 0.008). Laboratory findings revealed significantly lower levels of CRP (10.0 ± 11.6 vs. 37.6 ± 47.5 mg/L; *p* = 0.0005) and higher levels of hemoglobin (13.3 ± 2.2 vs. 12.3 ± 1.9 g/dL; *p* = 0.02) in chronic HF patients compared to solid tumor group. Differences in anthropometric variables and laboratory parameters, related to weight loss, between HF group and solid tumor group are given in Table 2.

**Table 2:** **Differences in anthropometric and laboratory variables, related to weight loss, between HF group and solid tumor group**.

	HF	Solid tumors	*p*-Value
Body weight (kg)	75.9 ± 15.4	73.4 ± 16.9	NS
Body mass index (kg/m^2^)	26.5 ± 4.8	24.4 ± 4.7	0.04*
Grip strength (kg)	17.2 ± 9.7	24.7 ± 9.6	0.008*
Mid-arm circumference (cm)	27.9 ± 3.7	28.4 ± 4.2	NS
Waist circumference (cm)	94.0 ± 11.2	94.8 ± 12.6	NS
Hip circumference (cm)	100.1 ± 8.8	99.0 ± 10.0	NS
Biceps skinfold (cm)	0.75 ± 0.44	0.66 ± 0.29	NS
Triceps skinfold (cm)	1.23 ± 0.50	1.11 ± 0.46	NS
Subscapular skinfold (cm)	1.43 ± 0.48	1.24 ± 0.54	0.04*
Suprailiac skinfold (cm)	1.28 ± 0.52	1.18 ± 0.60	NS
C-reactive protein (mg/L)	10.0 ± 11.6	37.6 ± 43.5	0.0005*
Hemoglobin (g/dL)	13.3 ± 2.2	12.3 ± 1.9	0.02*
Albumin (g/dL)	3.8 ± 0.5	3.5 ± 0.7	NS

*Values are given as a mean ± SD*.

*p < 0.05—Mann–Whitney U test.

*HF, heart failure; NS, not significant*.

### Anthropometric and Laboratory Findings—Chronic HF Patients vs. Solid Hematological Tumors Patients

When compared to patients with solid hematological tumors chronic HF patients were found to have lower muscle strength (17.2 ± 9.7 vs. 26.3 ± 10.2 kg; *p* = 0.0001) and mid-arm circumference (27.9 ± 3.7 vs. 31.1 ± 4.1 cm; *p* = 0.0004). Laboratory data showed that HF patients had lower levels of albumin (3.8 ± 0.5 vs. 4.1 ± 0.6 g/dL; *p* = 0.0008). Data for the two groups are shown in Table 3.

**Table 3:** **Differences in anthropometric and laboratory variables, related to weight loss, between HF group and solid hematological tumor group**.

	HF	Solid hematological tumors	*p*-Value
Body weight (kg)	75.9 ± 15.4	80.7 ± 18.0	NS
Body mass index (kg/m^2^)	26.5 ± 4.8	27.5 ± 5.5	NS
Grip strength (kg)	17.2 ± 9.7	26.3 ± 10.2	0.0001*
Mid-arm circumference (cm)	27.9 ± 3.7	31.1 ± 4.1	0.0004*
Waist circumference (cm)	94.0 ± 11.2	96.3 ± 14.9	NS
Hip circumference (cm)	100.1 ± 8.8	102.9 ± 9.7	NS
Biceps skinfold (cm)	0.75 ± 0.44	0.84 ± 0.35	NS
Triceps skinfold (cm)	1.23 ± 0.50	1.35 ± 0.45	NS
Subscapular skinfold (cm)	1.43 ± 0.48	1.50 ± 0.59	NS
Suprailiac skinfold (cm)	1.28 ± 0.52	1.49 ± 0.59	NS
C-reactive protein (mg/L)	10.0 ± 11.6	15.9 ± 36.1	NS
Hemoglobin (g/dL)	13.3 ± 2.2	12.7 ± 2.3	NS
Albumin (g/dL)	3.8 ± 0.5	4.1 ± 0.6	0.0008*

*Values are given as a mean ± SD*.

*p < 0.05—Mann–Whitney U test.

*HF, heart failure; NS, not significant*.

## Discussion

Weight loss is an independent predictor of mortality both in patients with cancer as well as in patients with chronic HF (11–13). It is a powerful predictor of adverse events during cancer chemotherapy (4) and surgical procedures (3).

Mechanisms of weight loss in patients with cancer and chronic HF are regarded similar (14). Numerous studies have revealed excessive elaboration of pro-inflammatory cytokines both in tumor patients and patients with HF. Those cytokines are responsible for exaggerated proteolysis and lipolysis as well as anorexia. All of these processes result in weight loss (1). Significant weight loss, called cachexia, is also a phenomenon observed in both HF and tumors as well as in some other chronic diseases. This advanced form of weight loss was also regarded as very similar in both conditions. Apparent similarity led to definitions of cachexia that were called generic, i.e., they could be applied to both patients with chronic HF and tumors (6). Yet, there was a general notion that a spectrum of metabolic and nutritional abnormalities of chronic diseases is wide and multifactorial in origin although some analogies exist (15). This notion led to newer, so called disease-specific definitions, of the cachexia syndrome. Cancer cachexia was the first nutritional disorder that was separately defined. New definition stated that it was designed for cancer patients and that the set criteria are cancer specific. It brought some other new concepts in studying the weight loss phenomenon most importantly the concept of staging of the weight loss (9). Concept of staging proved to have some benefits in subsequent clinical studies in cancer patients (16). This proof of some efficacy of new disease-specific definition and a general notion of multifactorial origin of weight loss in different diseases could not, in our opinion, substitute clinical studies. Such clinical studies should show that there are indeed some differences in presentations of patients with weight loss and different diseases. Yet such studies, that prove disease-specific weight loss concept, are generally lacking.

Certain experimental data led some authors to claim that “weight loss alone does not identify the full effect of cachexia on physical function and is not a prognostic variable” (17). This was a cornerstone to new definitions of cachexia which stated that aside from weight loss patient needs to fulfill additional clinical and laboratory criteria to be diagnosed as cachectic (6, 9). Some of those additional criteria like reduced muscle strength or increased inflammatory response (18, 19) were found to be independent predictors of mortality.

The lack of studies comparing patients with different diseases related to weight loss as well as significant influence of some additional parameters on survival of such patients were principal reasons of our study (20). We tried to find any significant differences in relevant anthropometric and laboratory features, related to weight loss, in patients with chronic HF compared to patients with various tumors and controls. We gathered data from 143 consecutive hospitalized patients with malignant diseases or chronic HF as well as data from 20 controls. Significant differences, both in anthropometric as well as laboratory features, in HF patients compared to control population were found. Those differences were not unexpected and were previously published (7). We then compared HF patients to patients with various tumor diseases. General notion of multifactorial nature of various cancers led us to further subdivide our tumor group. We decided to divide tumor patients into those with solid hematological tumors and those with solid tumors of any location. Initial analysis of amount of weight loss, intensity of weight loss, and prevalence of significant weight loss (>5% of weight in 12 months or less) found no differences between disease (i.e., HF vs. solid tumor vs. hematological solid tumor) groups. Analysis of anthropometric data revealed that patients with solid tumors had had lower values of BMI and subscapular skinfold thickness as well as higher muscle strength compared to HF group. On the other hand, laboratory findings revealed that HF patients had lower levels of CRP and higher levels of hemoglobin compared to solid tumor group. Compared to solid hematological tumor group, HF patients had lower levels of albumin, lower muscle strength, as well as lower mid-arm circumference.

Observed differences are strongly in favor of a new concept that looks at weight loss as a disease-specific phenomenon (9). Our results are first to our knowledge that directly prove such concept. They also imply different pathophysiological mechanisms of weight loss in patients with chronic HF compared to cancer patients. Although such mechanism are regarded as similar and probably rely mostly on excessive elaboration of pro-inflammatory cytokines found both in tumor patients and patients with HF (1), some differences have been found. Production of lipid mobilizing factor or proteolysis inducing factor was found exclusively in cancer patients (21) and could be a possible mechanisms responsible for the observed differences. There are also no studies that would compare profile and intensity of production of various inflammatory cytokines in patients with HF or cancer. Unfortunately, our study did not explore pathophysiological differences but could serve as a cornerstone for future studies addressing this issue.

While analyzing our data, we found several possible problems that could have confounded the results. First, HF patients were significantly older than both tumor groups as well as control group. This difference in age could in fact be responsible for some differences observed. It is known that age can influence both some anthropometric (22) as well as some laboratory (23, 24) measurements that were done in our study. One could say that observed strength reduction in HF patients could indeed be a reflection of age difference. On the other hand, some other observed differences, especially the ones in laboratory findings, cannot be explained by age differences. They are in fact quite opposite to what could be expected due to the age difference. Taking it all together, one can conclude that some part of our results could be influenced by age. On the other hand, most of the observed differences come probably from differences in pathophysiological mechanisms of various diseases included in our study and cannot be explained by age difference. Moreover, we believe that our results reflect clinical reality, i.e., the patients with HF are indeed older than tumor patients. If one is collecting data on consecutive patients, as we were, one should obtain the exact distribution of age as we did. To fully address the issue of age influence on our results, one should probably make an age-matched study. Our group is eagerly awaiting such results from other investigators.

Second possible problem could come from the fact that we collected data from hospitalized patients. This has given us a population of patients, especially in the solid tumor group, which does not reflect distribution of patients in general population. This specific distribution could have again influenced our results. Unfortunately, due to organizational/structural issues in our outpatient clinic during the timeframe of our interest, we gathered all data from hospitalized patients. In that way, the distribution of patients in our study reflects the population of patients hospitalized in our institution. Again we await results of others that would address that issue.

One must also bear in mind, that using anthropometric variables, especially skinfolds, in a heterogeneous population as ours, is not well validated. Those variables are well validated in general population and possible confounding could indeed exist. On the other hand, there have been no studies that have addressed this issue in systematical manner. Until such data are gathered, this confounding of our study, although possible, remains purely speculative.

We would also like to stress that the choice to divide patients in two groups as described earlier, lies in the organizational issues, i.e., those are the patients who are hospitalized in our institution. Patients with other diseases associated with weight loss, such as patients with obstructive pulmonary disease or renal disease, are less frequently encountered in our institution. Tumor patients were further subdivided into ones with solid tumors and ones with solid hematological tumors. The reason for that lies in the fact that solid hematological tumors usually do not result in such a pronounced weight loss (25). On the other hand, hematological patients are usually not in the scope of the interest in the studies investigating weight loss. Taking all this together, especially taking into account relatively high proportion of our patients belonging to the hematological group, one could speculate that such large hematology group could confound our data. To avoid that, we choose to divide our tumor group in such way.

Taken it all together, we strongly believe that observed differences are indicative of differences in mechanisms of weight loss between patients with HF and tumor patients. Further studies are warranted to fully elucidate possible pathophysiological differences and to address some possible pitfalls of our study. Till then, our results could serve as a cornerstone for future studies.

## Conclusion

There are differences in some anthropometric and laboratory measurements, related to weight loss, between patients with HF and patients with various tumor diseases. Observed differences could imply different mechanisms of weight loss in those groups of patients. Further studies, especially ones addressing pathophysiological mechanisms as well as addressing possible issues of our study, are warranted to confirm our findings.

## Ethics Statement

The study received previous local ethical board approval and was conducted according to the Declaration of Helsinki and its subsequent amendments. All patients gave written informed consent before participation in the study.

## Author Contributions

TL: gathered all the patients’ data and blood samples; contributed in the conception and design of the study, analysis and interpretation of data, critical revision and final approval of the version that is submitted. RV: contributed in the conception and design of the study, analysis and interpretation of data, critical revision and final approval of the version that is submitted in the design of the study and statistical analyses. ŽK: contributed in the conception and design of the study, analysis and interpretation of data, critical revision and final approval of the version that is submitted.

## Conflict of Interest Statement

The authors declare that the research was conducted in the absence of any commercial or financial relationships that could be construed as a potential conflict of interest.
